# Contrast Enhanced MRI in the Diagnosis of HCC

**DOI:** 10.3390/diagnostics5030383

**Published:** 2015-09-21

**Authors:** Eric Niendorf, Benjamin Spilseth, Xiao Wang, Andrew Taylor

**Affiliations:** Department of Diagnostic Radiology, University of Minnesota, 420 Delaware St SE, Minneapolis, MN 55455, USA; E-Mails: spil0042@umn.edu (B.S.); wangx962@umn.edu (X.W.); taylora@umn.edu (A.T.)

**Keywords:** MRI, HCC, Magnetic Resonance Imaging, Hepatocellular carcinoma, liver

## Abstract

Hepatocellular carcinoma (HCC) is the 6th most common cancer worldwide. Imaging plays a critical role in HCC screening and diagnosis. Initial screening of patients at risk for HCC is performed with ultrasound. Confirmation of HCC can then be obtained by Computed Tomography (CT) or Magnetic Resonance Imaging (MRI), due to the relatively high specificity of both techniques. This article will focus on reviewing MRI techniques for imaging HCC, felt by many to be the exam of choice for HCC diagnosis. MRI relies heavily upon the use of gadolinium-based contrast agents and while primarily extracellular gadolinium-based contrast agents are used, there is an emerging role of hepatobiliary contrast agents in HCC imaging. The use of other non-contrast enhanced MRI techniques for assessing HCC will also be discussed and these MRI strategies will be reviewed in the context of the pathophysiology of HCC to help understand the MR imaging appearance of HCC.

## 1. Introduction

Hepatocellular carcinoma (HCC) is the 6th most common cancer worldwide leading to the 3rd most likely cause of cancer related deaths [1,2]. The poor prognosis related to HCC development is illustrated by the fact that in most countries the mortality rate mirrors the incidence rate [3]. This malignancy is associated with chronic disease and cirrhosis in >90% of cases [3]. In Asia and Africa, hepatitis B virus is the most common cause for the chronically diseased liver while, in the West and Japan, it is the hepatitis C virus [4]. This difference in liver disease may lead to different results in HCC imaging, discussed later in this article. Other etiologies, such as alcohol abuse, hemochromatosis, and primary biliary cirrhosis, also contribute to chronically diseased liver and HCC. There is also rising concern about the present problem of obesity leading to non-alcoholic steatohepatitis and, subsequently, to cirrhosis with HCC development [5].

Imaging plays a critical role in HCC screening and diagnosis. Ultrasound is now the first and only screening exam for HCC in this patient population with the previously used serum alpha-fetoprotein now removed [6]. If a mass is questioned in screening ultrasound, a subsequent follow up imaging study is obtained. Presently, both contrast enhanced multidetector CT (MDCT) and MRI are options for further characterizing suspicious lesions detected by ultrasound [7]. Recently, strong advocates in the scientific community suggest that MRI, obtained with up-to-date techniques and in expert hands, is the exam of choice for HCC diagnosis [8,9,10]. Unlike most malignancies, which typically require biopsy for diagnosis, HCC can be diagnosed based on CT or MRI characteristics alone due to the relatively high specificity of these modalities [11]. Although the imaging diagnosis of HCC using MDCT/MRI is not without some concerns [12,13], the complications, complexities, ambiguity of diagnosis, and cost of biopsy confirmation are considered too great [7,14]. Once the imaging diagnosis is made, therapy is chosen based on extent of disease, liver function, and patient age and comorbidities. Thus, imaging for the presence and extent of HCC in chronically diseased liver is crucial for patient-management options, which include liver transplantation or various locoregional or systemic therapies.

This article will review the critical role of intravenous gadolinium contrast in MRI performed for HCC detection and quantification of disease extent. It is important that the specifics of contrast enhanced MRI series acquisition be understood for the greatest appreciation of the remainder of this article. Multiple post-contrast imaging series are obtained to provide a reflection of the vascularity/neovascularity of tissue and allow improved specificity and sensitivity in the diagnosis of HCC. A typical MRI protocol includes a 3D T1-weighed fat saturated sequence before administering intravenous contrast and at three points in time following contrast administration. The first of three post-contrast phases is the “late arterial phase”. Timing of the late arterial phase (typically between 25 and 30 s post contrast injection) is critical for detection of HCC with intravascular contrast present in the hepatic artery and the portal vein, but no contrast in the hepatic veins. Next is the “portal venous” phase (typically 65 to 70 s post contrast injection), in which there is dense contrast enhancement in the portal vein, but the hepatic veins also contain contrast. Finally, the third “delayed” or “equilibrium” phase is obtained typically 3 min from start of injection.

Chronic liver disease results in the development of regenerative nodules; proliferating hepatic cells cordoned off by variously thickened strands of fibrous tissue. A very small fraction of these nodules may dedifferentiate into premalignant and eventually malignant lesions [15,16]. In the typical pathway, a regenerative nodule progresses first to a dysplastic nodule (low grade then possibly high grade), then to a well-differentiated HCC, and finally to a moderately/poorly differentiated HCC. As dedifferentiation progresses, there is neoangiogenesis that replaces the normal hepatic parenchymal portal venous inflow with recruited, abnormal, arterial vessels [17]. This neoangiogenesis results in a fairly predictable decrease in portal vein inflow and increase in hepatic artery inflow. The worse the tumor grade, the greater the neovascular development typically becomes [18,19]. The presence of this neoplastic arterial system is unusual in the dysplastic nodule and is extremely rare in the regenerative nodule [20]. Coupled with this is the tendency for the well-differentiated HCC to be smaller in diameter than the more aggressive tumors; the well differentiated tumors usually being ≤2 cm in diameter [21]. Therefore, as a rule, larger tumors (>2 cm) are more likely to be of higher tumor grade and more likely to demonstrate tumor neovascularity and increased arterial flow. Additionally, in hepatocarcinogenesis, the dedifferentiating cells themselves undergo changes that imaging will be able to take advantage of in categorizing. There is worsening of cellular atypia changes with increasing nuclear-to-cytoplasmic ratio within each cell, thickening of the cellular plate, and increase in the abnormal hepatocyte density. When this is combined with the associated loss of the extracellular space, the diffusivity of water molecules decreases [15,22]. This local anatomy will lead to decrease in water mobility and can result in abnormal Diffuse Weighted Imaging (DWI) characteristics.

Thus, arterial enhancement on dynamic imaging, as well as increased signal visualized on DWI, will help differentiate the collection of cells in these different stages. Regenerative nodules and low-grade dysplastic nodules will not have either of these imaging findings. However, high grade dysplastic nodules, which can be histologically difficult to separate out from a well-differentiated HCC [23], may occasionally show arterial enhancement and increased signal intensity on DWI sequences [24,25].

However not all tumors demonstrate the typical pattern of neovascularity and some HCCs, including the poorer grades, will only be visualized in the post arterial phases, *i.e.*, the portal venous or delayed phase [21,26,27]. Blood flow in HCCs typically passes through the lesion and into the venous outflow more rapidly than normal hepatic parenchyma, leading to a characteristic “washout” appearance of relative hypoenhancement of the tumor relative to the liver on these phases 88% of the time. Further, 97% of moderately/poorly differentiated tumors are appreciated in this phase [21].

**Figure 1:**
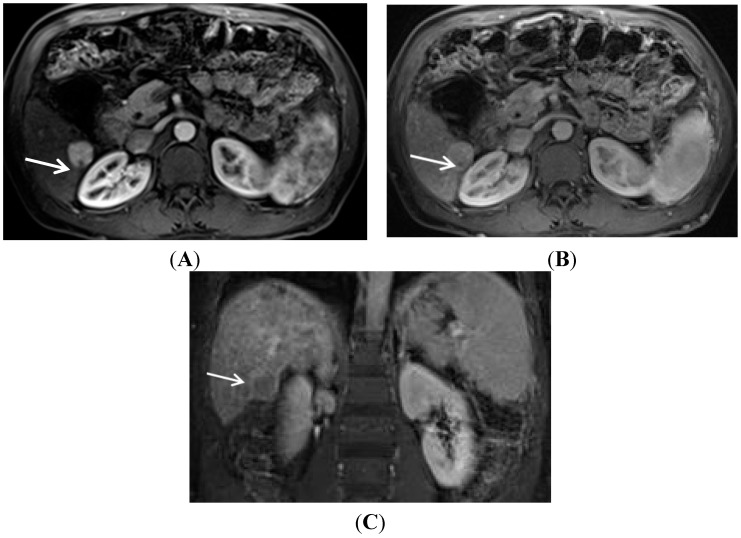
Typical contrast enhancement for MRI diagnosis of HCC. (**A**) On a late arterial phase image, a 2.3 cm HCC (arrow) demonstrates unequivocal arterial enhancement relative to the surrounding liver; (**B**) On the portal venous phase of the same exam, the hepatocellular carcinoma (arrow) has decreased in enhancement relative to the earlier arterial phase while the hepatic parenchyma has increased in signal intensity; (**C**) A coronal image obtained in the equilibrium phase demonstrates the hepatocellular carcinoma (arrow) as being of lower signal intensity than the adjacent cirrhotic parenchyma (“washout”). Additionally present is the delayed capsule or so-called pseudo-capsule, which helps further solidify the imaging diagnosis of hepatocellular carcinoma.

The characteristic enhancement of the regenerative nodule to HCC progression has allowed for the development of specific criteria in HCC diagnosis. “Hyperenhancement” of the tumor in the late arterial phase is very important for the diagnosis of HCC [28,29,30]. This “hyperenhancement” is defined as unequivocal increased enhancement of the lesion relative to the surrounding liver. However, other etiologies can have hyperenhancement in the arterial phase, such as nontumoral “vascular blush” associated with altered vascular flow or shunting, cholangiocarcinoma, which can also be seen in chronically diseased liver, and multiple other liver lesions, such adenomas or the common hepatic hemangioma or focal nodular hyperplasia. In addition, dysplastic nodules, which have not yet progressed to HCC, can rarely demonstrate hyperenhancement. Therefore, the addition of hypointensity, or “washout”, on the portal venous phase and/or equilibrium phase is used to separate out these other causes from the true HCC hyperenhancement. The combination of arterial hyperenhancement followed by washout in the portal venous and/or delayed phase has a sensitivity of 64%–89%; specificity of 96%; and positive predictive value of 93% for the diagnosis of HCC [29,31,32]. At present, both the American Association for the Study of Liver Diseases (AASLD) and the United Network for Organ Sharing (UNOS) systems use the dimension of a nodule >1 cm having arterial hyperenhancement followed by washout to qualify for the diagnosis of HCC [6,33]. There is also the potential development of an enhancing rim surrounding the HCC on delayed imaging called a capsule or pseudocapsule [34]. This finding adds to the strength of diagnosis for HCC. The presence of this capsule is critical in allowing the diagnosis of HCC to an otherwise suspicious nodule ≥1 cm and <2 cm in diameter according to the Organ Procurement and Transplantation Network (OPTN) Classification [27]. This pattern of arterial enhancement, followed by “washout” and pseudocapsule formation is demonstrated in Figure 1.

**Figure 2:**
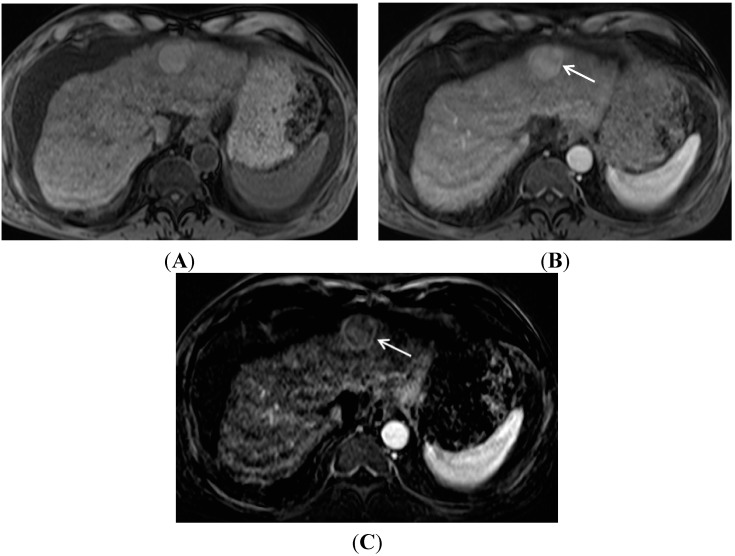
Importance of image subtraction in display of enhancement. (**A**) A T1 hyperintense hepatic nodule (arrow) is present on the pre-contrast image; (**B**) A late arterial phase gadolinium enhanced T1 gradient echo image demonstrates a bright nodule (arrow) suspicious for arterial enhancement; (**C**) On the subtraction image, the true, mild, enhancement of the nodule (arrow) is apparent.

While the presence of enhancement and washout is critical for HCC diagnosis, clinical assessment of changes in enhancement are often complicated by underlying heterogeneous signal in chronically diseased livers, which may be due to the presence of nodules, hepatic steatosis and/or hemosiderosis. Some hepatic nodules may be of increased signal intensity in precontrast T1-weighted imaging, due to the presence of fat, copper, mucus, or proteins [35]. In this circumstance, the impression of contrast enhancement may be uncertain unless careful comparison between the precontrast and late arterial phase contrast images is performed. Generating subtracted imaging series (subtraction of each post-contrast imaging series from the pre-contrast series) can help assessment of these cases by subtracting out pre-contrast signal “heterogeneity” and yielding an imaging series which better demonstrates true enhancement (Figure 2). However, subtracted imaging series require a reproducible breath-holding technique for satisfactory series co-registration, and can be degraded by motion artifacts [36].

Beyond diagnosis, imaging provides insight into the prognosis of management of a patient’s tumor load, as well as defining whether there is malignant venous involvement or not. One major use of MR imaging is evaluating patients for liver transplant candidacy, which, under current OPTN guidelines, requires that tumor burden be within threshold criteria. The Milan criteria is the most commonly used gauge for defining the amount of tumor burden for this purpose. For a patient to be within the Milan criteria, and thus remain a candidate for liver transplant, there must be imaging proof of the absence of vascular invasion and metastatic disease, and presence of (1) three or less HCC lesions, all ≤3 cm in diameter; or (2) one HCC ≤5 cm in diameter [37].

MRI is critical in evaluating for the presence of venous tumor thrombus, which is more frequently portal venous but also occasionally hepatic venous. The presence of tumor thrombus typically excludes transplant and drastically changes treatment. While diagnosing tumor thrombus can be complex and numerous factors may be helpful in differentiating tumor thrombus from bland thrombus, a major criteria used for determining thrombus is the presence of arterial phase enhancement, which is expected to be identified in malignant thrombus but not bland thrombus [38,39]. The presence of arterial enhancement indicating malignant thrombus is often most easily distinguished on subtracted post-contrast series [36,38,39].

## 2. Gadolinium Contrast Agent Selection

As discussed above, an intravenous contrast agent is a critical component for HCC imaging. The contrast agent almost universally used is a gadolinium chelate complex. There are other contrast agents available but which are not in common clinical use, such as superparamagnetic iron oxide (ferumoxides, Feridex^®^, Bayer HealthCare, Berlin, Germany; ferucarbotran, Resovist^®^, Bayer HealthCare), and a manganese-based agent, MnDPDP (no longer available in the U.S.).

The element gadolinium is a potent paramagnetic ion able to cause a decrease in both T1 and T2 relaxation times, thereby altering signal intensity on MR imaging sequences. At concentrations seen in the intravascular and tissue interstitial compartments, the gadolinium contrast agent will affect T1 relaxation, leading to an increased signal intensity, “enhancing”, structure on T1 weighted sequences. It is this increased signal intensity or “enhancement”, which is critical in HCC imaging. However, when highly concentrated, such as in the urinary tract, gadolinium-induced effects on the T2 relaxation time can predominate, and can cause loss of signal intensity on both T1 and T2 weighted imaging sequences.

The gadolinium-based contrast agents (GBCA) commonly used clinically for hepatic imaging can be divided into two categories: Extracellular and hepatobiliary. Extracellular gadolinium-based contrast agents (for example, gadopentetate dimeglumine (Gd-DTPA), Magnevist^®^, Bayer HealthCare), in widespread use today, are small molecules that course from the vascular space into the interstitial compartment. Approximately 95% of this extravascular gadolinium agent is excreted via glomerular filtrate in an unchanged state. This extracellular gadolinium contrast agent has four different biochemical structures: Macrocyclic and linear, both available in ionic or non-ionic forms. The details concerning the advantages and disadvantages of each contrast category is beyond the scope of this article, but in general there is little clinical difference [40]. With normal renal function, this extravascular agent has a half-life of approximately 1.5 h [41] with greater than 95% excretion in 24 h [42]. The standard dose is 0.1 mmol/kg typically injected intravenously at a rate of 2 mL/s followed by a normal saline “flush” of 20 to 50 mL.

The second category is the “hepatobiliary” gadolinium contrast agent. These agents have both an interstitial distribution and, importantly for hepatic imaging, they also have hepatocyte uptake with subsequent biliary excretion. Temporally, the first agent available was gadobenate dimeglumine (MultiHance, Gd-BOPTA, Bracco Diagnostics, Princeton, NJ, USA), approved for use in the United States in 2004. Approximately 95% of this agent is excreted by the kidney, but 3% to 5% is taken up by the normal hepatocytes and excreted into the biliary tract [43]. Because of the relatively small amount of hepatic uptake, imaging in the “hepatobiliary phase” (HBP), where the liver parenchyma is of increased signal intensity, is from 45 to 120 min post contrast injection. In practice, most institutions image this second phase at 1 to 2 h. The dosing and injection rate is similar to that of the extracellular agents, although, on certain occasions, a half dose of 0.05 mmol/kg can be used.

In 2008, a second hepatobiliary agent, gadoxetic acid (U.S.: Eovist; Europe: Primovist, Gd-EOB-DTPA, Bayer Healthcare Pharmaceuticals, Wayne, NJ, USA) was approved for use in the U.S. This agent has approximately 50:50 excretion between renal (glomerular filtration) and hepatocyte uptake/biliary excretion. Gadoxetic acid can therefore be used for the early dynamic imaging phase in the liver, as above, followed by a 20 min T1-weighted imaging phase where the liver is brighter and nonhepatocyte containing masses will be variably dark. This hepatobiliary agent was initially approved by the FDA for a dose of 0.025 mmol/kg. However, some studies suggest better results with 0.05 mmol per kilogram, which is now a very common practice pattern [43]. Note that even with this “double dose” of gadoxetic acid, the patient will receive half the gadolinium dose compared with the standard dose of the other GBCAs.

**Figure 3:**
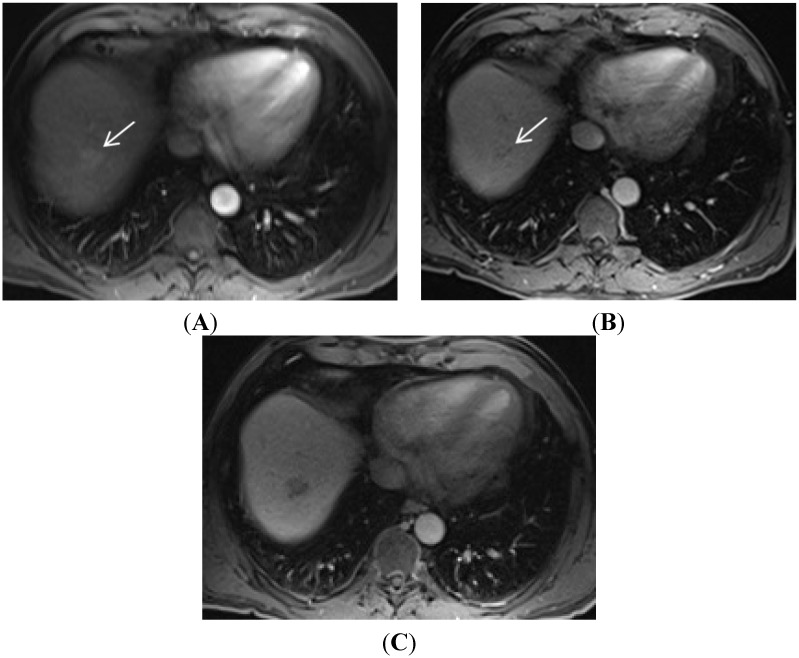
The importance of gadoxetic acid for visualization of small and/or ill-defined HCC. (**A**) A subtle area of vague enhancement (arrow) is seen in the late arterial phase sequence in this patient with end-stage liver disease; (**B**) This area (arrow) on the portal venous phase remains ill-defined and could easily be missed; (**C**) The 20-min hepatobiliary phase of this gadoxetic acid contrast enhanced exam demonstrates a well-defined area of low signal intensity. This image allows critical review of the corresponding area on the earlier sequences to now appreciate the minimal hyperenhancement in the arterial phase and the very subtle washout of the portal venous phase to suggest a diagnosis of HCC.

**Figure 4:**
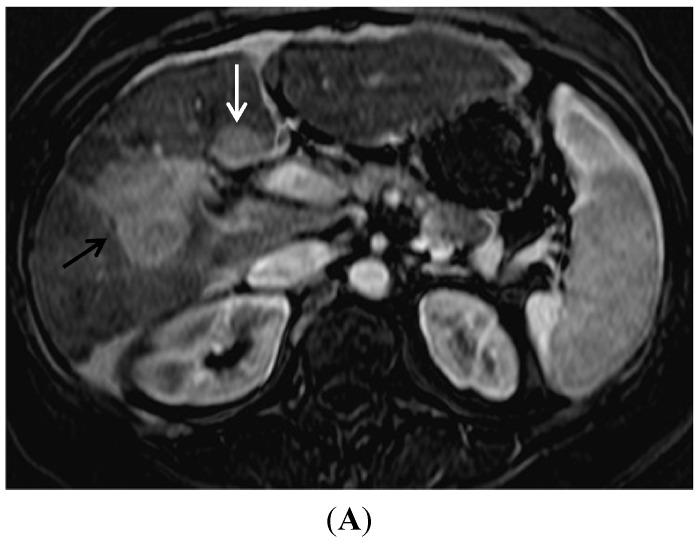

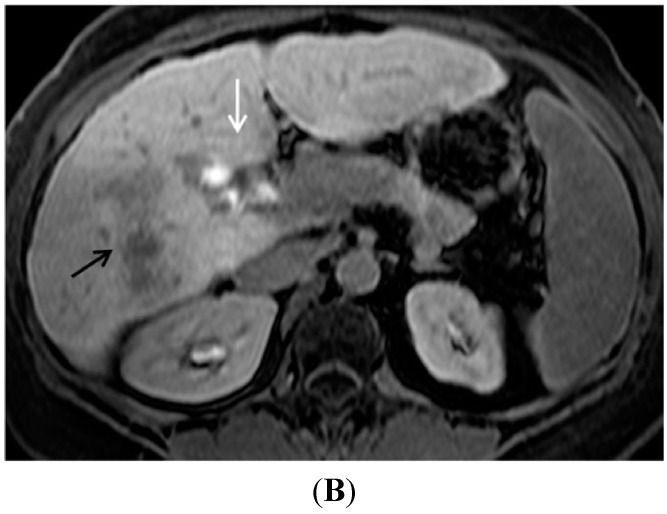
Gadoxetic acid contrast distinguising benign arterial hyperenhancement of cirrhosis *versus* HCC. (**A**) On the subtracted late arterial phase Gadoxetic acid enhanced study there are 2 areas of hyperenhancement (black and white arrows); (**B**) The hepatobiliary phase from the same exam the shows the low signal area of the HCC (black arrow) but the normal hepatic uptake and therefore normal signal intensity in hepatic segment IV (white arrow), consistent with benign vascular shunting. Subsequent exams (not shown) demonstrated the loss of the segment IV arterial contrast enhancement with the continued presence of normal hepatic uptake in this area.

**Figure 5:**
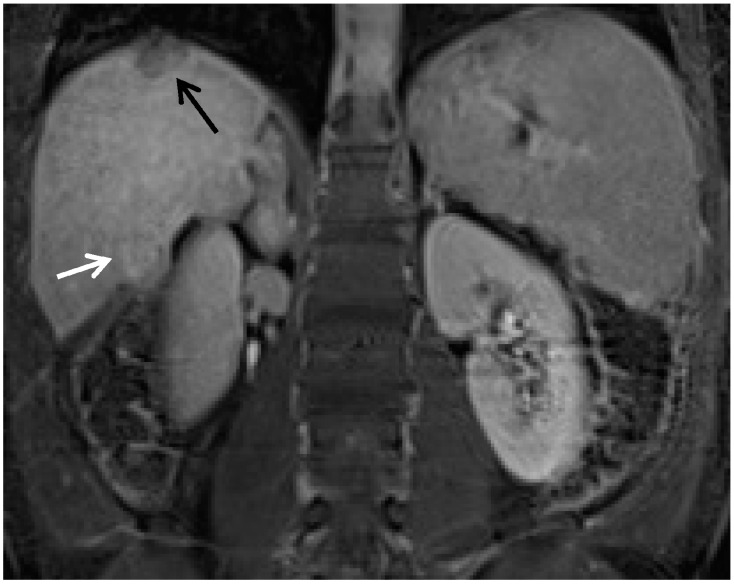
Uptake of gadoxetic acid in a hepatocellular carcinoma. This coronal image was obtained 15 min after intravenous injection of Gadoxetic acid. (Note that this exam was obtained by a combination contrast technique. This was obtained by first injecting an extracellular gadolinium agent and imaging through the equilibrium phase. This was followed by Gadoxetic acid injection with subsequent hepatobiliary imaging at 15 min.) There are two HCC’s present on this image. The lesion at the dome (black arrow) demonstrates a well circumscribed, hypointense area compatible with the mass without normally functioning hepatocytes. This mass had arterial hyperenhancement and delayed phase washout. These findings are all compatible with hepatocellular carcinoma. The lesion in segment 5–6 (white arrow) is also a hepatocellular carcinoma (same patient as in Figure 1 but it demonstrates uptake of the hepatobiliary agent.

The use of hepatobiliary agents for the diagnosis of HCC is in transition. Some major HCC imaging guidelines do not mention this class of GBCA [14,32,44], while other societies or organizations include their use [45,46]. Many authors and consensus panels are suggesting various uses for the hepatobiliary agents for HCC workup [47,48]. The HBP can be used for increasing the sensitivity of HCC detection of small lesions, help suggest the diagnosis of HCC in lesions without washout, help diagnosis of HCC in lesions without arterial hyperenhancement, and differentiate arterial pseudolesions from HCC (Figure 3, Figure 4 and Figure 5) [10,48]. However, while hepatobiliary phase imaging has been helpful to increase sensitivity, it has not yet been incorporated into OPTN and AASLD guidelines as a way to establish the definitive diagnosis of HCC. It remains unclear whether hepatobiliary phase contrast hypoenhancement will be able to be incorporated in a way that is as specific as conventional extracellular contrast criteria for HCC diagnosis.

Issues related to the uptake of the hepatobiliary contrast agent in some HCCs, the lack of delayed phase “pseudo-capsule”, which is available in the extracellular agents, and respiratory motion during the arterial phase of dynamic imaging are all potential problems at present [47]. Additionally, with worsening of cirrhosis, the ability of HCC detection decreases, probably because of poorer contrast uptake leading to lower contrast enhancement [10,47]. It has also been variably reported that between 9% and 20% of typical HCCs can take up the hepatobiliary agent and become iso- or hyperintense on the hepatobiliary phase, which creates obvious problems with HCC detection [43,49,50,51]. However, some authors suggest that this hepatobiliary agent uptake in HCCs present in the West may be less of a problem [52].

## 3. Other MRI Sequences for HCC and in Relation to GBCA

The standard, contrast-enhanced, hepatic MRI exam has other sequences that can be helpful in HCC diagnosis: T2 weighted imaging, diffusion weighted imaging, and T1 weighted chemical shift “in and out of phase” imaging. The information obtained from these sequences can be important in support of the diagnosis of HCC, but is not used in the major criteria for HCC diagnosis, which is obtained through the contrast-enhanced criteria cited above.

**Figure 6:**
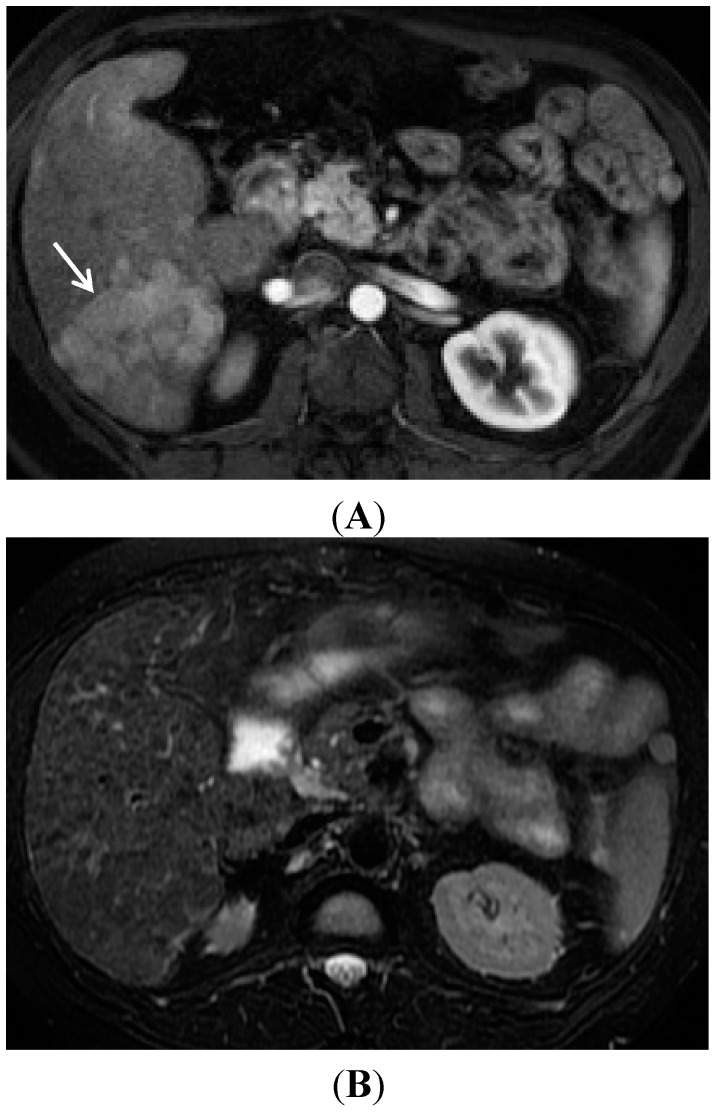
The potential unreliable display of HCC on T2W imaging. (**A**) A late arterial phase contrast enhanced image nicely demonstrates the large HCC (arrow) by moderate contrast enhancement; (**B**) However a technically excellent fat saturated T2W image very poorly demonstrates any textural difference in this area. At most there is slightly lower signal intensity in this area and a subtle loss of the normal hepatic texture.

In the 1990s up to the mid-2000s, T2 weighted imaging was considered to be very helpful for HCC visualization in cirrhotic liver, but it has now fallen out of favor [53,54]. Unfortunately, accuracy of T2 is limited as not all HCC display the typical increased signal intensity on T2 weighted sequences (for example Figure 6) and not all increased signal intensity mass lesions are HCC. As the dynamic contrast imaging sequences using T1 weighted gradient echoes has become much more robust, the T2 imaging sequence has lost importance. However, lesions that contain iron are often seen as hypointense relative to liver on T2 weighted sequences. This can be useful if present, because in HCC the tumor cells lose the ability to concentrate iron, therefore T2 hypointensity essentially excludes the diagnosis of HCC [55].

Diffusion weighted imaging (DWI) is another, more recent, advancement of MRI HCC detection. This MRI technique uses the difference in water molecule movement to help detect and characterize liver masses. The contrast in this sequence stems from water movement in the microenvironment. When the water molecules are not allowed to diffuse freely, two symmetric gradients with opposite direction applied during a T2 echo planar spin echo sequence will result in increased signal intensity. Malignancies typically do not allow free water movement and therefore result in restriction and thus hyperintensity. The DWI sequence uses different strengths of bipolar gradients, *i.e.*, different *b*-values, to create different imaging characteristics in the same local environment. Using the sequence of DWI alone, high *b*-value (600–1000) imaging demonstrates a moderate sensitivity, approximately 70%, but also a high specificity for HCC detection [22,56,57]. The high specificity associated with high *b*-value DWI is very helpful in distinguishing HCC from arterially enhancing pseudolesions and other benign cirrhotic lesions [22]. Rarely, however, a benign cirrhotic nodule may demonstrate DWI hyperintensity, thought possibly to be related to edematous change or infarct [22,58,59]. It should be noted however that with a worsening of cirrhotic background changes, there is a decrease in the ability of DWI to define an HCC [60].

At present, DWI is typically used in conjunction with contrast enhancement. Here, it is shown to improve detection rates, especially related to small lesions (<2 cm). One study increased detection rate of small HCC’s from 60% to 85% [61], while another study increased the HCC detection sensitivity from 85% to 98% when used with contrast enhanced MRI using an extracellular contrast agent [62]. Finally, DWI can be of help with gadoxetic acid studies as well. When combined with DWI hyperintensity, the likelihood of HCC diagnosis is increased when a nodule may only show arterial hyperenhancement alone or hypointensity on the hepatobiliary phase alone [63].

A final ancillary finding that can be displayed using T1 weighted “in and out of phase” chemical shift imaging is the presence of intralesional fat. This imaging sequence utilizes resonance frequency differences between water and fat to acquire a set of images when fat and water are in phase (or additive) and a second set of images when fat and water or out of phase (which results in signal cancellation) to demonstrate the presence of microscopic intravoxel fat. Fatty metamorphosis associated with HCC is found in up to 17% of HCCs, and therefore can be helpful in discriminating between HCC and other lesions that do not contain fat [46,64,65].

## 4. Conclusions

In summary, current MRI techniques and criteria for diagnosing hepatocellular carcinoma have evolved to rely most heavily upon the use of gadolinium-based contrast agents. Current established criteria for diagnosing HCC are based primarily on the extracellular properties of contrast agents, however there is an emerging role of hepatobiliary contrast agents in HCC imaging. Ancillary sequences, such as DWI, T2 weighted imaging, and T1 weighted chemical shift imaging, play a supportive role in the diagnosis of HCC but are not major diagnostic criteria. Subtracted post-contrast imaging series can be helpful for more accurately assessing hepatic lesion enhancement characteristics and also for assessing vascular involvement.

## References

[1] Baron R.L. (2014). The radiologist as interpreter and translator. Radiology.

[2] Ferlay J., Shin H.R., Bray F., Forman D., Mathers C., Parkin D.M. (2010). Estimates of worldwide burden of cancer in 2008: Globocan 2008. Int. J. Cancer.

[3] Bruix J., Gores G.J., Mazzaferro V. (2014). Hepatocellular carcinoma: Clinical frontiers and perspectives. Gut.

[4] Willatt J.M., Hussain H.K., Adusumilli S., Marrero J.A. (2008). Mr imaging of hepatocellular carcinoma in the cirrhotic liver: Challenges and controversies. Radiology.

[5] Chalasani N., Younossi Z., Lavine J.E., Diehl A.M., Brunt E.M., Cusi K., Charlton M., Sanyal A.J. (2012). The diagnosis and management of non-alcoholic fatty liver disease: Practice guideline by the American gastroenterological association, American association for the study of liver diseases, and American college of gastroenterology. Gastroenterology.

[6] Organ Procurement and Transplantation Network Allocation of Livers. http://optn.transplant.hrsa.gov/ContentDocuments/OPTN_Policies.pdf#nameddest=Policy_09.

[7] Choi J.Y., Lee J.M., Sirlin C.B. (2014). CT and MR imaging diagnosis and staging of hepatocellular carcinoma: Part II. Extracellular agents, hepatobiliary agents, and ancillary imaging features. Radiology.

[8] Vauthey J.N., Dixon E., Abdalla E.K., Helton W.S., Pawlik T.M., Taouli B., Brouquet A., Adams R.B., American Hepato-Pancreato-Biliary Association, Society of Surgical Oncology (2010). Pretreatment assessment of hepatocellular carcinoma: Expert consensus statement. HPB.

[9] Lee Y.J., Lee J.M., Lee J.S., Lee H.Y., Park B.H., Kim Y.H., Han J.K., Choi B.I. (2015). Hepatocellular carcinoma: Diagnostic performance of multidetector CT and MR imaging—A systematic review and meta-analysis. Radiology.

[10] Sirlin C.B., Hussain H.K., Jonas E., Kanematsu M., Min Lee J., Merkle E.M., Peck-Radosavljevic M., Reeder S.B., Ricke J., Sakamoto M. (2014). Consensus report from the 6th international forum for liver MRI using gadoxetic acid. J. Magn. Reson. Imaging.

[11] Bruix J., Sherman M., Practice Guidelines Committee, American Association for the Study of Liver Diseases (2005). Management of hepatocellular carcinoma. Hepatology.

[12] Bialecki E.S., Ezenekwe A.M., Brunt E.M., Collins B.T., Ponder T.B., Bieneman B.K., di Bisceglie A.M. (2006). Comparison of liver biopsy and noninvasive methods for diagnosis of hepatocellular carcinoma. Clin. Gastroenterol. Hepatol..

[13] Crippin J.S. (2006). Biopsy of suspicious liver nodules: Does it change management?. Clin. Gastroenterol. Hepatol..

[14] European Association for the Study of the Liver, European Organisation for Research and Treatment of Cancer (2012). EASL-EORTC clinical practice guidelines: Management of hepatocellular carcinoma. J. Hepatol..

[15] Coleman W.B. (2003). Mechanisms of human hepatocarcinogenesis. Curr. Mol. Med..

[16] Efremidis S.C., Hytiroglou P. (2002). The multistep process of hepatocarcinogenesis in cirrhosis with imaging correlation. Eur. Radiol..

[17] Matsui O. (2004). Imaging of multistep human hepatocarcinogenesis by CT during intra-arterial contrast injection. Intervirology.

[18] Hayashi M., Matsui O., Ueda K., Kawamori Y., Kadoya M., Yoshikawa J., Gabata T., Takashima T., Nonomura A., Nakanuma Y. (1999). Correlation between the blood supply and grade of malignancy of hepatocellular nodules associated with liver cirrhosis: Evaluation by CT during intraarterial injection of contrast medium. Am. J. Roentgenol..

[19] Tajima T., Honda H., Taguchi K., Asayama Y., Kuroiwa T., Yoshimitsu K., Irie H., Aibe H., Shimada M., Masuda K. (2002). Sequential hemodynamic change in hepatocellular carcinoma and dysplastic nodules: CT angiography and pathologic correlation. AJR Am. J. Roentgenol..

[20] Park Y.N., Yang C.P., Fernandez G.J., Cubukcu O., Thung S.N., Theise N.D. (1998). Neoangiogenesis and sinusoidal “Capillarization” in dysplastic nodules of the liver. Am. J. Surg. Pathol..

[21] Monzawa S., Ichikawa T., Nakajima H., Kitanaka Y., Omata K., Araki T. (2007). Dynamic CT for detecting small hepatocellular carcinoma: Usefulness of delayed phase imaging. Am. J. Roentgenol..

[22] Lim K.S. (2014). Diffusion-weighted MRI of hepatocellular carcinoma in cirrhosis. Clin. Radiol..

[23] International Working Party (1995). Terminology of nodular hepatocellular lesions. Hepatology.

[24] Hussain S.M., Reinhold C., Mitchell D.G. (2009). Cirrhosis and lesion characterization at MR imaging. Radiographics.

[25] Lee M.H., Kim S.H., Park M.J., Park C.K., Rhim H. (2011). Gadoxetic acid-enhanced hepatobiliary phase MRI and high-b-value diffusion-weighted imaging to distinguish well-differentiated hepatocellular carcinomas from benign nodules in patients with chronic liver disease. Am. J. Roentgenol..

[26] Hwang G.J., Kim M.J., Yoo H.S., Lee J.T. (1997). Nodular hepatocellular carcinomas: Detection with arterial-, portal-, and delayed-phase images at spiral CT. Radiology.

[27] Choi B.I., Lee H.J., Han J.K., Choi D.S., Seo J.B., Han M.C. (1997). Detection of hypervascular nodular hepatocellular carcinomas: Value of triphasic helical CT compared with iodized-oil CT. Am. J. Roentgenol..

[28] Shinmura R., Matsui O., Kobayashi S., Terayama N., Sanada J., Ueda K., Gabata T., Kadoya M., Miyayama S. (2005). Cirrhotic nodules: Association between MR imaging signal intensity and intranodular blood supply. Radiology.

[29] Marrero J.A., Hussain H.K., Nghiem H.V., Umar R., Fontana R.J., Lok A.S. (2005). Improving the prediction of hepatocellular carcinoma in cirrhotic patients with an arterially-enhancing liver mass. Liver Transpl..

[30] Torimura T., Ueno T., Kin M., Harada R., Taniguchi E., Nakamura T., Sakata R., Hashimoto O., Sakamoto M., Kumashiro R. (2004). Overexpression of angiopoietin-1 and angiopoietin-2 in hepatocellular carcinoma. J. Hepatol..

[31] Valls C., Andía E., Sánchez A., Moreno V. (2003). Selective use of low-osmolality contrast media in computed tomography. Eur. Radiol..

[32] Bruix J., Sherman M., American Association for the Study of Liver Diseases (2011). Management of hepatocellular carcinoma: An update. Hepatology.

[33] Lee J.M., Yoon J.H., Joo I., Woo H.S. (2012). Recent advances in CT and MR imaging for evaluation of hepatocellular carcinoma. Liver Cancer.

[34] Ishigami K., Yoshimitsu K., Nishihara Y., Irie H., Asayama Y., Tajima T., Nishie A., Hirakawa M., Ushijima Y., Okamoto D. (2009). Hepatocellular carcinoma with a pseudocapsule on gadolinium-enhanced MR images: Correlation with histopathologic findings. Radiology.

[35] Furlan A., Marin D., Bae K.T., Lagalla R., Agnello F., Bazzocchi M., Brancatelli G. (2009). Focal liver lesions hyperintense on T1-weighted magnetic resonance images. Semin. Ultrasound CT MRI.

[36] Yu J.S., Rofsky N.M. (2003). Dynamic subtraction mr imaging of the liver: Advantages and pitfalls. Am. J. Roentgenol..

[37] Mazzaferro V., Regalia E., Doci R., Andreola S., Pulvirenti A., Bozzetti F., Montalto F., Ammatuna M., Morabito A., Gennari L. (1996). Liver transplantation for the treatment of small hepatocellular carcinomas in patients with cirrhosis. N. Engl. J. Med..

[38] Sandrasegaran K., Tahir B., Nutakki K., Akisik F.M., Bodanapally U., Tann M., Chalasani N. (2013). Usefulness of conventional MRI sequences and diffusion-weighted imaging in differentiating malignant from benign portal vein thrombus in cirrhotic patients. Am. J. Roentgenol..

[39] Shah Z.K., McKernan M.G., Hahn P.F., Sahani D.V. (2007). Enhancing and expansile portal vein thrombosis: Value in the diagnosis of hepatocellular carcinoma in patients with multiple hepatic lesions. Am. J. Roentgenol..

[40] Bellin M.F. (2006). MR contrast agents, the old and the new. Eur. J. Radiol..

[41] Oksendal A.N., Hals P.A. (1993). Biodistribution and toxicity of MR imaging contrast media. J. Magn. Reson. Imaging.

[42] Tweedle M.F. (1997). The prohance story: The making of a novel MRI contrast agent. Eur. Radiol..

[43] Frydrychowicz A., Lubner M.G., Brown J.J., Merkle E.M., Nagle S.K., Rofsky N.M., Reeder S.B. (2012). Hepatobiliary MR imaging with gadolinium-based contrast agents. J. Magn. Reson. Imaging.

[44] Wald C., Russo M.W., Heimbach J.K., Hussain H.K., Pomfret E.A., Bruix J. (2013). New OPTN/UNOS policy for liver transplant allocation: Standardization of liver imaging, diagnosis, classification, and reporting of hepatocellular carcinoma. Radiology.

[45] Kudo M., Izumi N., Kokudo N., Matsui O., Sakamoto M., Nakashima O., Kojiro M., Makuuchi M., HCC Expert Panel of Japan Society of Hepatology (2011). Management of hepatocellular carcinoma in japan: Consensus-based clinical practice guidelines proposed by the Japan Society of Hepatology (JSH) 2010 updated version. Dig. Dis..

[46] LI-RADS v2014. http://nrdr.acr.org/lirads/.

[47] Motosugi U., Bannas P., Sano K., Reeder S.B. (2015). Hepatobiliary MR contrast agents in hypovascular hepatocellular carcinoma. J. Magn. Reson. Imaging.

[48] Hope T.A., Fowler K.J., Sirlin C.B., Costa E.A., Yee J., Yeh B.M., Heiken J.P. (2015). Hepatobiliary agents and their role in LI-RADS. Abdom. Imaging.

[49] Tsuboyama T., Onishi H., Kim T., Akita H., Hori M., Tatsumi M., Nakamoto A., Nagano H., Matsuura N., Wakasa K. (2010). Hepatocellular carcinoma: Hepatocyte-selective enhancement at gadoxetic acid-enhanced MR imaging—Correlation with expression of sinusoidal and canalicular transporters and bile accumulation. Radiology.

[50] Kitao A., Matsui O., Yoneda N., Kozaka K., Kobayashi S., Koda W., Gabata T., Yamashita T., Kaneko S., Nakanuma Y. (2012). Hypervascular hepatocellular carcinoma: Correlation between biologic features and signal intensity on gadoxetic acid-enhanced MR images. Radiology.

[51] Kim J.Y., Kim M.J., Kim K.A., Jeong H.T., Park Y.N. (2012). Hyperintense HCC on hepatobiliary phase images of gadoxetic acid-enhanced MRI: Correlation with clinical and pathological features. Eur. J. Radiol..

[52] Bashir M.R., Gupta R.T., Davenport M.S., Allen B.C., Jaffe T.A., Ho L.M., Boll D.T., Merkle E.M. (2013). Hepatocellular carcinoma in a north american population: Does hepatobiliary MR imaging with Gd-EOB-DTPA improve sensitivity and confidence for diagnosis?. J. Magn. Reson. Imaging.

[53] Hussain H.K., Syed I., Nghiem H.V., Johnson T.D., Carlos R.C., Weadock W.J., Francis I.R. (2004). T2-weighted MR imaging in the assessment of cirrhotic liver. Radiology.

[54] Hecht E.M., Holland A.E., Israel G.M., Hahn W.Y., Kim D.C., West A.B., Babb J.S., Taouli B., Lee V.S., Krinsky G.A. (2006). Hepatocellular carcinoma in the cirrhotic liver: Gadolinium-enhanced 3D T1-weighted MR imaging as a stand-alone sequence for diagnosis. Radiology.

[55] Choi J.Y., Lee J.M., Sirlin C.B. (2014). CT and MR imaging diagnosis and staging of hepatocellular carcinoma: Part I. Development, growth, and spread: Key pathologic and imaging aspects. Radiology.

[56] Muhi A., Ichikawa T., Motosugi U., Sano K., Matsuda M., Kitamura T., Nakazawa T., Araki T. (2009). High-b-value diffusion-weighted MR imaging of hepatocellular lesions: Estimation of grade of malignancy of hepatocellular carcinoma. J. Magn. Reson. Imaging.

[57] Motosugi U., Ichikawa T., Sou H., Sano K., Tominaga L., Muhi A., Araki T. (2010). Distinguishing hypervascular pseudolesions of the liver from hypervascular hepatocellular carcinomas with gadoxetic acid-enhanced mr imaging. Radiology.

[58] Kim J.E., Kim S.H., Lee S.J., Rhim H. (2011). Hypervascular hepatocellular carcinoma 1 cm or smaller in patients with chronic liver disease: Characterization with gadoxetic acid-enhanced MRI that includes diffusion-weighted imaging. AJR Am. J. Roentgenol..

[59] Vandecaveye V., de Keyzer F., Verslype C., Op de Beeck K., Komuta M., Topal B., Roebben I., Bielen D., Roskams T., Nevens F. (2009). Diffusion-weighted MRI provides additional value to conventional dynamic contrast-enhanced MRI for detection of hepatocellular carcinoma. Eur. Radiol..

[60] Kim A.Y., Kim Y.K., Lee M.W., Park M.J., Hwang J., Lee M.H., Lee J.W. (2012). Detection of hepatocellular carcinoma in gadoxetic acid-enhanced MRI and diffusion-weighted MRI with respect to the severity of liver cirrhosis. Acta Radiol..

[61] Piana G., Trinquart L., Meskine N., Barrau V., Beers B.V., Vilgrain V. (2011). New MR imaging criteria with a diffusion-weighted sequence for the diagnosis of hepatocellular carcinoma in chronic liver diseases. J. Hepatol..

[62] Xu P.J., Yan F.H., Wang J.H., Lin J., Ji Y. (2009). Added value of breathhold diffusion-weighted MRI in detection of small hepatocellular carcinoma lesions compared with dynamic contrast-enhanced MRI alone using receiver operating characteristic curve analysis. J. Magn. Reson. Imaging.

[63] Park M.J., Kim Y.K., Lee M.W., Lee W.J., Kim Y.S., Kim S.H., Choi D., Rhim H. (2012). Small hepatocellular carcinomas: Improved sensitivity by combining gadoxetic acid-enhanced and diffusion-weighted mr imaging patterns. Radiology.

[64] Martín J., Sentís M., Zidan A., Donoso L., Puig J., Falcó J., Bella R. (1995). Fatty metamorphosis of hepatocellular carcinoma: Detection with chemical shift gradient-echo MR imaging. Radiology.

[65] Basaran C., Karcaaltincaba M., Akata D., Karabulut N., Akinci D., Ozmen M., Akhan O. (2005). Fat-containing lesions of the liver: Cross-sectional imaging findings with emphasis on MRI. AJR Am. J. Roentgenol..

